# Numerical Simulation on Solidification during Vertical Centrifugal Casting Process for TC4 Alloy Wheel Hub with Enhanced Mechanical Properties

**DOI:** 10.3390/ma17010184

**Published:** 2023-12-29

**Authors:** Yujie Yang, Xiangyi Wang, Xiangming Li, Rongfeng Zhou, Zhengyuan He, Yehua Jiang

**Affiliations:** 1 School of Materials Science and Engineering, Kunming University of Science and Technology, Kunming 650093, China; m18513629115@163.com (Y.Y.); a1137357069@163.com (X.W.); lixm@kust.edu.cn (X.L.); zhourfchina@hotmail.com (R.Z.); jiangyehua@kmust.edu.cn (Y.J.); 2 National-Local Joint Engineering Research Center for Technology of Advanced Metallic Solidification Forming and Equipment, Kunming 650093, China

**Keywords:** TC4 alloy, vertical centrifugal casting, numerical simulation, microstructure, mechanical properties

## Abstract

The Ti-6Al-4V (TC4) alloy wheel hub has exhibited some defects that affect the properties during the vertical centrifugal casting process. Therefore, the analysis of the solidification process would contribute to solving the above-mentioned problems. In this study, an orthogonal experimental design was employed to optimize the process parameters (rotational speed, mold preheating temperature, and pouring temperature) of the vertical centrifugal casting method. The effects of process parameters on the velocity field, temperature field, and total shrinkage porosity during the solidification process were explored, and the microstructure and mechanical properties of the wheel hub prepared by the vertical centrifugal casting method were also investigated. The results showed that the rotational speed mainly induced the change of the velocity field. The pouring temperature and mold preheating temperature affected the temperature field and solidification time. Based on the analysis of the orthogonal experiment, the optimal parameters were confirmed as a rotational speed of 225 rpm, mold preheating temperature of 400 °C, and pouring temperature of 1750 °C, respectively. The simulation results of total shrinkage porosity were in agreement with the experiment results. The wheel hub was composed of nonuniform *α* and *β* phases. The lath *α* phase precipitated from larger *β* grains with different orientations. Compared with the other samples at different locations, the *α* phase in the PM sample (middle of the TC4 wheel hub) displayed high peak intensity and uniformly distributed *β* phase along the radial direction of the wheel hub. Moreover, the PM sample revealed a higher tensile strength of 820 MPa and similar Vickers hardness of 318 HV compared with the other samples at different locations, which were higher than those of rolling and extrusion molding. This experiment design would provide a good reference for the vertical centrifugal casting of the TC4 alloy.

## 1. Introduction

Ti-6Al-4V (TC4) alloy, as a two-phase (*α* + *β*) titanium alloy, displayed remarkable structural stability, excellent mechanical properties, high plasticity, toughness, and resistance to high-temperature deformation [1,2,3]. Due to these characteristics, the TC4 alloy was extensively applied in various areas, such as compressor blades and impellers of jet engines, landing gear wheels, aircraft accessories, and pipes [4,5,6]. Various complex TC4 alloy parts were commonly fabricated through methods such as casting, forging, and machining [7,8,9,10]. Interestingly, both the forging method (877 MPa) and the casting method (836 MPa) exhibited similar tensile strengths for the TC4 alloy [11]. As a result, the casting method aroused significant interest due to the short process and low cost [12,13]. Notably, the vertical centrifugal casting method, as one of the precision casting technologies, could further enhance the filling capacity of liquid metal through centrifugal force [14,15].

During the solidification process in vertical centrifugal casting, the complex forces and movements of liquid metal could lead to defects. Generally, the numerical simulation method helped researchers gain a better understanding and analysis of the solidification process of liquid metal in mold cavities during casting. It could also predict defects like shrinkage porosity, which was crucial for the adjustment of process parameters and the quality control of castings. It was reported that the shrinkage porosity of node castings was successfully eliminated to predict the formation of shrinkage porosity by simulating the solidification processes [16]. Thus, the yield of TC4 large cylinder liner castings was significantly improved [17]. It was well known that the casting quality was effectively enhanced by optimized process parameters [18,19]. The process parameters, such as mold temperature, pouring temperature, and centrifugal speed, were thoroughly evaluated and optimized by the simulation of the solidification process, which would further improve the mechanical properties of the prepared TC4 castings [20,21].

Based on the above considerations, the process parameters, e.g., pouring temperature, mold preheating temperature, and rotational speed, were designed through an orthogonal test to improve the forming quality of the TC4 wheel hub. The velocity field, temperature field, and defect distribution in the solidification process were systematically simulated by ProCAST 2018 software during the vertical centrifugal casting process. Subsequently, the microstructure and mechanical properties of the TC4 wheel hub prepared by the vertical centrifugal casting method were also characterized.

## 2. Materials and methods

### 2.1. Orthogonal Experimental Design

The experimental design and data analysis utilized the orthogonal experimental method. Three process parameters included rotational speed (rpm), mold preheating temperature (°C), and pouring temperature (°C), all of which could have an impact on shrinkage during vertical centrifugal casting.

To evaluate the effects of these parameters, a nine-degree-of-freedom orthogonal array was selected, which contained three different levels for each parameter, as shown in Table 1 [22,23].

Subsequently, the nine combined parameters were input into ProCAST 2018 software to simulate the solidification process of the TC4 wheel hub prepared by vertical centrifugal casting. The quantitative scoring criterion was the ratio of the number of grids containing defects to the total number of grids in the wheel hub. Range analysis was then employed to assess the influence of different factors and levels.

### 2.2. Numerical Simulation

**Figure 1:**
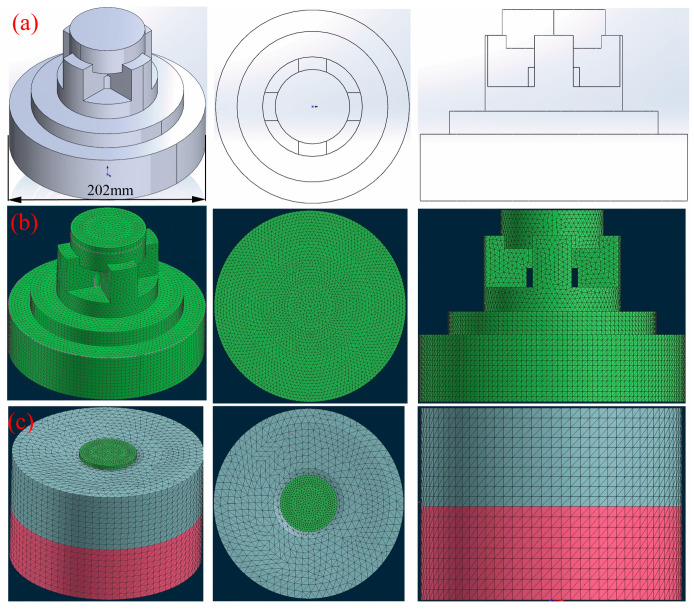
Three-dimensional model diagrams of TC4 wheel hub: (**a**) geometric model; (**b**) mesh model; (**c**) mesh model. The blue part of the figure shows the upper mold, the red part is the lower mold, and the green part is the casting of the hub.

The three-dimensional model diagram of the TC4 wheel hub was established by SolidWorks software, as depicted in Figure 1.

The model underwent pre-processing of the mesh module, where the mesh was divided into the surface mesh and body mesh. The mesh size of the graphite mold was set to the default value of 10 mm, while the casting was set to 4 mm. Subsequently, the direction of gravity, filling rate, and a host of other boundary conditions were determined in the CAST module and checked by the “check” function of ProCAST. Finally, the numerical simulation was executed after thorough verification. Additional simulation parameters are summarized in Table 2.

The flow phenomena of liquid metal during mold filling in the vertical centrifugal casting process were very complex. It could be considered an unsteady flow of an incompressible viscous fluid with free surface with respect to a rotational frame of reference. Its motion state could be described by the equations of momentum (Equation (1)) and mass conservation (Equation (2)) [24]:

(1) $$\frac{\partial }{\partial t}\left(\rho \vec{u}\right)+\nabla .\left(\vec{\rho }\right)=\nabla .\left(\mu \nabla \vec{u}\right)-\nabla P+S$$

(2) $$\begin{array}{c} \frac{\partial \rho }{\partial t}+\nabla .\left(\rho \vec{u}\right)=0 \end{array}$$

where $\vec{u}$, *ρ*, *μ*, and *P* denoted the velocity vector, density, dynamic viscosity, and pressure. The vector source term *S* in the above equation represents the body forces and influence of boundaries.

Fluid configurations were defined in terms of a volume of fluid (VOF) function *F*(*x*, *y*, *z*, *t*) and was governed by the following advection (Equation 3):

(3) $$\begin{array}{c} \frac{\partial F}{\partial t}+\nabla \left(\vec{u}F\right)=0 \end{array}$$

For a single fluid, *F* represents the volume fraction occupied by the fluid. Thus, the fluid existed where *F* = 1, and void regions correspond to locations where *F* = 0. In the directional solidification process, the metal pouring and solidification of the wheel hub took place in a vacuum environment. The heat transfer process of the directional solidification process could be described by the law of conservation of energy as in Equation (4):

(4) $$\begin{array}{c} \rho C_{p}\frac{\partial \left(T\right)}{\partial \left(t\right)}=\nabla \left(\lambda \nabla \left(T\right)\right)+Q_{M}+Q_{R} \end{array}$$

where *T* was temperature; *t* was time; *ρ* was the density; C*_p_* was the specific heat of the material; *λ* was the thermal conductivity; *Q_M_* was an internal heat source; and *Q_R_* was the heat flux density between the surface element and the environment. During the solidification process, the liquid metal released latent heat, and the internal heat source could be expressed as in Equation (5):

(5) $$\begin{array}{c} Q_{M}=\rho ∆H\frac{\partial \left(f_{s}\right)}{\partial \left(t\right)} \end{array}$$

where Δ*H* was the latent heat, and *f_s_* was the volume fraction of the solid phase.

### 2.3. Preparation of the Wheel Hub

The casting process of the wheel hub involved the use of a vacuum self-consuming electrode arc shell condensing furnace (LEYBOLD.AG, L300SM, Cologne, Germany). The chemical compositions of the TC4 wheel hub were analyzed by ICP-OES (Shimadzu, ICPE-9820, Kyoto, Japan), as shown in Table 3. 

**Figure 2:**
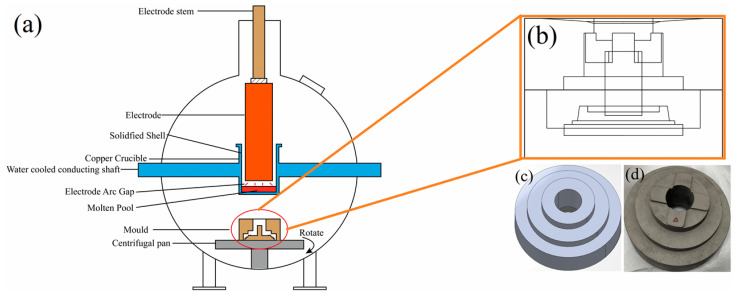
Schematic diagram of (**a**) vacuum self-consuming electrode arc shell condensing furnace, (**b**) the casting mold, (**c**) the mold of wheel hub, (**d**) As-cast Ti-6Al-4V wheel hub.

A schematic diagram of the vacuum self-consuming electrode arc shell condensing furnace is shown in Figure 2a. In this process, the molten electrode of TC4 alloy bar flowed into a water-cooled copper crucible with a layer of condensate shell. It effectively prevented contamination of the liquid metal from the crucible. The graphite mold, as shown in Figure 2b, was positioned on the centrifugal pan.

A sufficient amount of liquid metal was poured into the graphite mold and held for 5 min at a constant speed. The wheel hub model and casting are shown in Figure 2c,d. The rotational speed, mold preheating temperature, and pouring temperature were set according to the process parameters of orthogonal experiments. Finally, the wheel hubs prepared using Level 5 and Level 6 parameters were selected to verify reliability of the numerical simulation results, respectively.

### 2.4. Microstructure and Mechanical Properties Characterization

The samples from the inside, middle, and outside of the wheel hub were cut down and labeled as PI, PM, and PO, respectively. The microstructures of samples undergoing grinding, polishing, etching, and cleaning were observed by a metallographic microscope (Nikon, MA200, Tokyo, Japan). Subsequently, more detailed observations were conducted using scanning electron microscopy (SEM, ZEISS EVO18, Oberkochen, Germany) at a voltage of 5–10 kV. In addition, the element distribution on the sample surface was qualitatively analyzed using an energy dispersive spectrometer (EDS). Furthermore, the phases of the samples were investigated by X-ray diffraction (XRD, Bruker D8 Advance, Karlsruhe, Germany). The diffraction angle ranged from 20 to 80°, and the scanning speed was 5°/min.

The tensile samples with size of 30 × 5 × 1 mm were machined by wire cutting machine. The tensile tests were conducted using a universal mechanical testing machine (MTS, E45-205, Eden Prairie, MN, USA). A total of 5 tensile samples at each position were measured. Microhardness of the samples was examined using an automatic micro-Vickers hardness tester (Shimadzu, HMV-G-FA-S, Kyoto, Japan) under a load of 4.9 N with a holding time of 10 s.

## 3. Results and Discussion

### 3.1. Numerical Simulation Results and Analysis

In order to improve the mechanical properties of the wheel hub, the pouring temperature, mold preheating temperature, and rotational speed were optimized, and the effects of these factors on the microstructure and mechanical properties of the wheel hub were carefully characterized.

**Figure 3:**
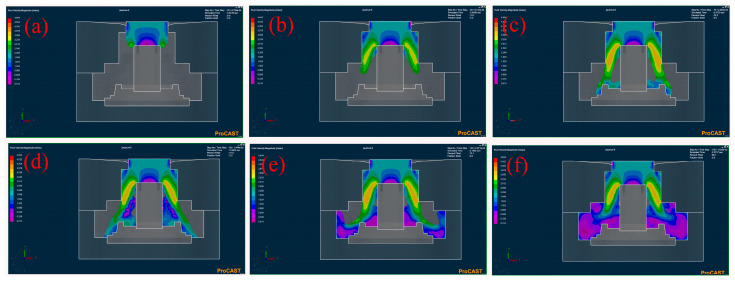
Velocity field of numerical simulation at different times of the filling stage for the Level 5 (R225M400P1750): percentage filled of 9.6 (**a**), 19.7 (**b**), 32.2 (**c**), 41 (**d**), 70.1 (**e**), and 98.1 (**f**).

The velocity field at different times during the filling process for Level 5 was demonstrated, as shown in Figure 3. The overall fill time of the wheel hub was approximately 0.23 s (pouring speed: 35 kg/s). As shown in Figure 3a, in the initial stage of the filling process, the liquid metal flowed down vertically under gravity. The velocity of the partial liquid metal decreased after contact with the mold (0.014 m/s), and the other part continued to flow toward the lower sprue, as shown in Figure 3b. At this time, the velocity of the liquid metal (3.234 m/s) continued to increase under the action of gravity and centrifugal force. After contacting the bottom of the mold, the liquid metal filled from inside to outside perpendicular to the axis of rotation, as shown in Figure 3c,d. When the velocity of the liquid metal decreased, the part near the inner mold was not completely filled due to the centrifugal force (Figure 3e,f). It could be deduced that the centrifugal force induced a trend of outward movement of the liquid metal [25].

**Figure 4:**
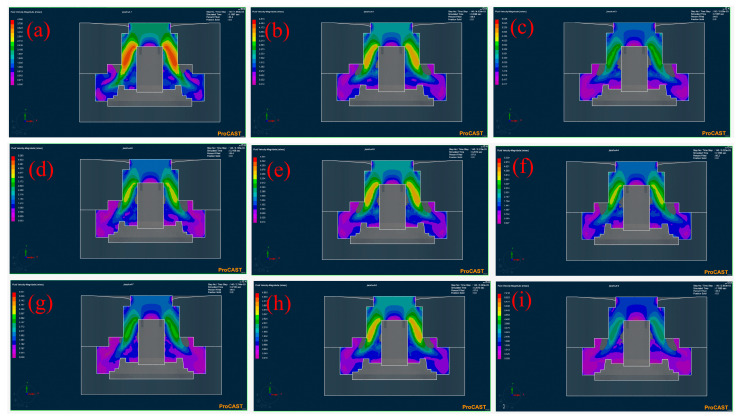
Velocity field of numerical simulation when the percentage filled is approximately 85%. (**a**) Level 1: R200M400P1730, (**b**) Level 2: R225M450P1730, (**c**) Level 3: R250M500P1730, (**d**) Level 4: R200M500P1750, (**e**) Level 5: R225M400P1750, (**f**) Level 6: R250M450P1750, (**g**) Level 7: R200M450P1770, (**h**) Level 8: R225M500P1770, (**i**) Level 9: R250M400P1770.

When the filled percentage was approximately 85%, the simulated velocity field of the wheel hub under all experimental protocols was shown in Figure 4. It could be observed that the unfilled area of the wheel hub was mainly concentrated in the middle area near the molds, probably due to the rotational speed. The effect of mold preheating temperature and rotational speed on the velocity field could be observed through the comparison of Levels 1, 2, and 3 (Figure 4a–c). It could be seen that the maximum velocity of the liquid metal increased, and the unfilled area closed to the inner mold expanded as the mold’s preheating temperature and rotational speed increased. Compared to Levels 1, 5, and 9 (Figure 4a,e,i), it could also be seen that the maximum velocity of the liquid metal increased with the increased rotational speed and pouring temperature, and the unfilled area near the inside of the mold expanded. In addition, there was no significant change under the maximum rotational speed among Levels 2, 5, and 8 (Figure 4b,e,h). It indicated that the rotational speed as a main factor affected the maximum velocity of liquid metal. That is to say, the defect distribution of the wheel hub could be affected by the velocity field of the liquid metal. Although high rotational speed increased the filling rate of liquid metal, too high of a rotational speed could significantly increase the content of inclusions in liquid metal [20], which was not conducive to the casting quality.

**Figure 5:**
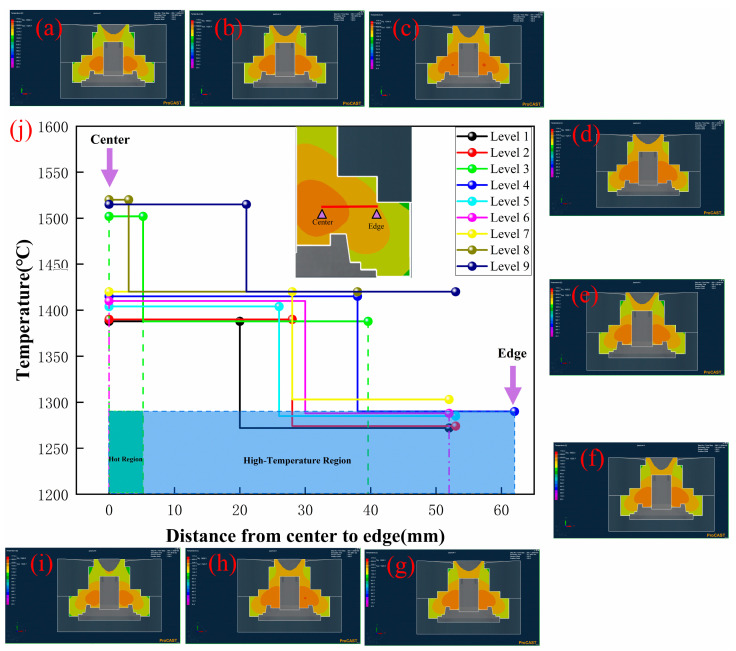
Temperature field of numerical simulation at 160 s and temperature graph of casting sample from center to edge: (**a**) Level 1: R200M400P1730, (**b**) Level 2: R225M450P1730, (**c**) Level 3: R250M500P1730, (**d**) Level 4: R200M500P1750, (**e**) Level 5: R225M400P1750, (**f**) Level 6: R250M450P1750, (**g**) Level 7: R200M450P1770, (**h**) Level 8: R225M500P1770, (**i**) Level 9: R250M400P1770. (**j**) Wheel hub temperature graph of high-temperature regions.

The temperature field distribution of the wheel hub at the solidification time of 160 s is illustrated in Figure 5. Under different process parameters, the temperature field distribution exhibited some common characteristics: the temperature of the casting significantly decreased from center to edge (Figure 5j). As the inner and outer surfaces of the casting contacted the graphite mold, the heat transfer rate became faster, resulting in a gradient downward trend of the casting temperature from center (1380–1520 °C) to edge (1050–1200 °C). Compared with Levels 2, 5, and 8 (Figure 5b,e,h) in the orthogonal experiments under the same rotational speed, the temperature field still exhibited some differences, where the wheel hub of Level 8 displayed a hot region. Therefore, it could be inferred that the rotational speed had almost no effect on the temperature field [26]. 

The effect of mold preheating temperature on the temperature field was observed through the comparison of Levels 1–3 (Figure 5a–c) in an orthogonal experiment. When the mold’s preheating temperature increased from 400 to 500 °C, the high-temperature region of the wheel hub became larger, and the hot region about 1500 °C also appeared in Level 3 (Figure 5c). The higher preheating temperature would decrease the temperature difference between the wheel hub and the graphite mold, which would induce a slower cooling rate. Similarly, when the pouring temperature increased from 1730 to 1770 °C under the same mold preheating temperature, the hot region (1520 °C) of Level 9 (Figure 5i) was higher than those of Level 1 and 5 (Figure 5a,e), and the high-temperature region (1400 °C) of Level 9 (Figure 5i) was larger than those of Level 1 and 5. Therefore, the higher the pouring temperature, the longer the cooling time of the wheel hub. It could manifest a reduction in the cooling rate [27]. It was also deduced that the pouring temperature and mold preheating temperature had a significant effect on the temperature field: the temperature of the hot region and the area of the high-temperature region had an effect on the cooling rate of the liquid metal [28].

**Figure 6:**
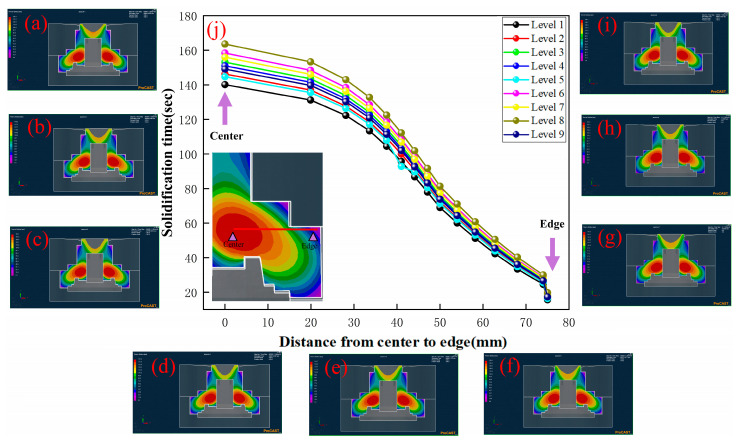
Solidification time of numerical simulation: (**a**) Level 1: R200M400P1730, (**b**) Level 2: R225M450P1730, (**c**) Level 3: R250M500P1730, (**d**) Level 4: R200M500P1750, (**e**) Level 5: R225M400P1750, (**f**) Level 6: R250M450P1750, (**g**) Level 7: R200M450P1770, (**h**) Level 8: R225M500P1770, (**i**) Level 9: R250M400P1770. (**j**) Solidification time of the sample from center to edge.

The solidification times of the wheel hub from the hot region to the outer surface are shown in Figure 6.

It exhibited some common characteristics: the solidification time tended to decrease from the center to the edge, which was aligned with the temperature change trend depicted in Figure 5. When the pouring temperature was kept constant, the increase in the mold preheating temperature resulted in the longer solidification time of the wheel hub, observed by the comparison of Level 1 (Figure 6a) to Level 3 (Figure 6c). It was attributed to the decrease in temperature difference between the wheel hub and the graphite mold caused by the higher preheating temperature, thus leading to a decrease in the cooling rate. Compared with Level 1 (Figure 6a), Level 5 (Figure 6e), and Level 9 (Figure 6i), it could be concluded that higher pouring temperatures resulted in longer solidification times of the wheel hub under the same mold preheating temperature. This could be explained by the similar effect of pouring temperature on the temperature field discussed in the previous section, where an increase in pouring temperature led to a longer solidification time. Considering the analysis conclusions of the temperature field, as shown in Figure 6j, the three groups (Level 1, Level 2, and Level 5) with the shorter solidification time exhibited a lower temperature hot region, smaller high-temperature region, and faster cooling rates (Figure 5j). Therefore, it could be inferred that the mold preheating temperature and pouring temperature affected the solidification time so as to induce the defect formation of the wheel hub due to the lower cooling rate. It has been reported [29] that the hot regions with slower cooling rates due to higher temperatures were highly susceptible to defects, such as shrinkage and shrinkage holes. Therefore, the cooling rate should also be taken into account, as well as the selection of the mold preheating temperature and pouring temperature. The lower mold preheating temperature and appropriate pouring temperature would meet the fluidity of liquid metal to avoid defects caused by the hot region.

**Figure 7:**
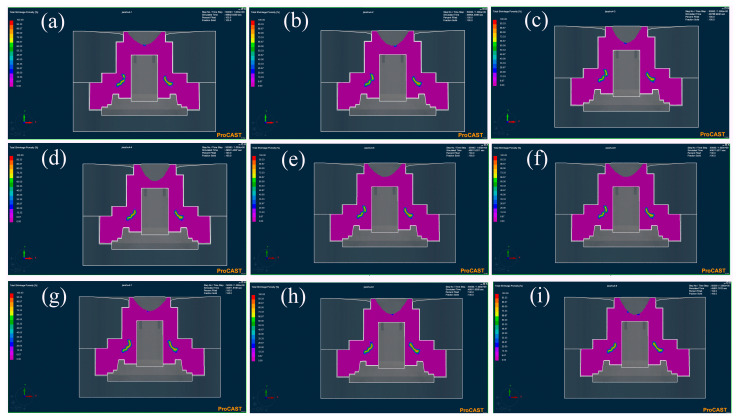
The total shrinkage porosity of numerical simulation: (**a**) Level 1: R200M400P1730, (**b**) Level 2: R225M450P1730, (**c**) Level 3: R250M500P1730, (**d**) Level 4: R200M500P1750, (**e**) Level 5: R225M400P1750, (**f**) Level 6: R250M450P1750, (**g**) Level 7: R200M450P1770, (**h**) Level 8: R225M500P1770, (**i**) Level 9: R250M400P1770.

**Figure 8:**
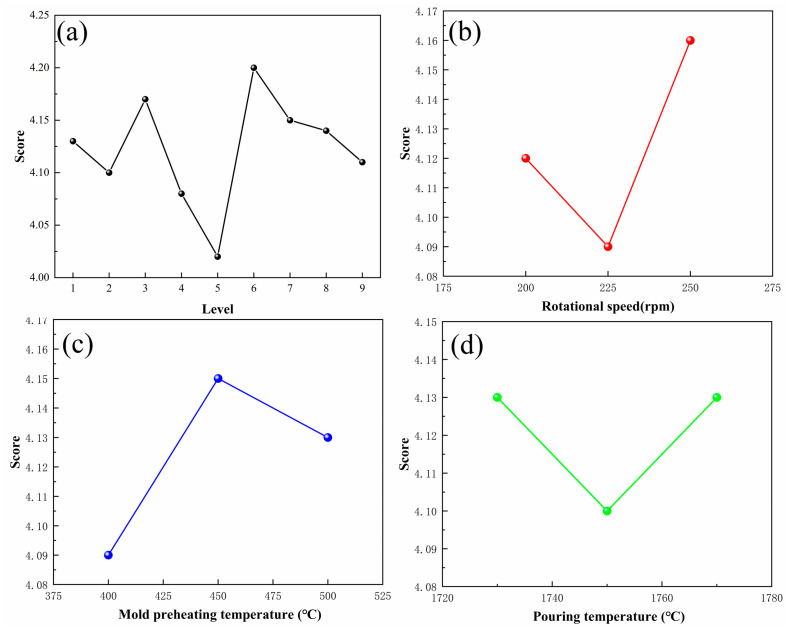
The statistical chart of scores in orthogonal experiment. Statistical chart of (**a**) total shrinkage porosity, (**b**) rotational speed, (**c**) mold preheating temperature, and (**d**) pouring temperature.

The distribution of the total shrinkage porosity represented the defects distribution of casting, as shown in Figure 7. It was observed that the defects were primarily concentrated in the center of the wheel hubs. This location was similar to the high-temperature region in the temperature field diagram. The statistical score of the orthogonal experiment is shown in Figure 8 and Table 4. It revealed that Level 5 had the fewest defects, with a score of 4.02, while Level 6 had the most defects, with a score of 4.20. The K value represents the average score of one factor in each level. It indicates the influence degree of a different factor on casting quality. The r value represents the variance of each factor. The larger r value revealed a greater effect on the casting. The rotational speed of K2 < K1 < K3 exhibited the best value of 225 rpm. Similarly, we obtained the best mold preheating temperature of 400 °C and the best-pouring temperature of 1750 °C according to the lowest K value. Compared with the mold preheating temperature and pouring temperature, the rotational speed with the biggest r value of 0.07 exhibited the highest influence on the casting quality. 

**Figure 9:**
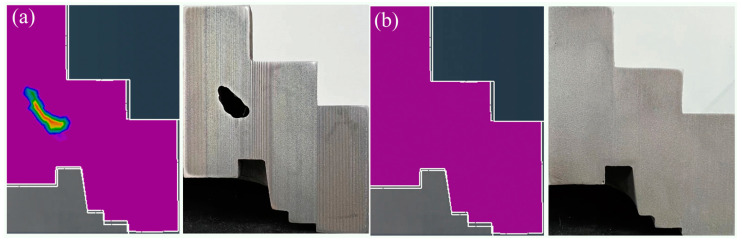
Comparative analysis between numerical simulation and experiment: (**a**) defects for Level 6, (**b**) non-defects for Level 5. The purple areas in the figure are areas of lower shrinkage porosity, and the closer the color tends to red, the higher the shrinkage porosity of the wheel hub.

So, to summarize, the recommended process parameters were as follows: rotational speed of 225 rpm, mold preheating temperature of 400 °C, and pouring temperature of 1750 °C. Furthermore, a vertical centrifugal casting process according to these process parameters (Level 5 and Level 6) was implemented to prepare the TC4 wheel hubs. Figure 9 shows the experimental result and simulation results of the castings. It could be seen that the middle of the wheel hub revealed defects in both the simulation results and the experimental results for Level 6 (Figure 9a), whereas there were no such defects for Level 5 (Figure 9b). It indicated that the experimental results were consistent with the simulation results.

### 3.2. Microstructure

**Figure 10:**
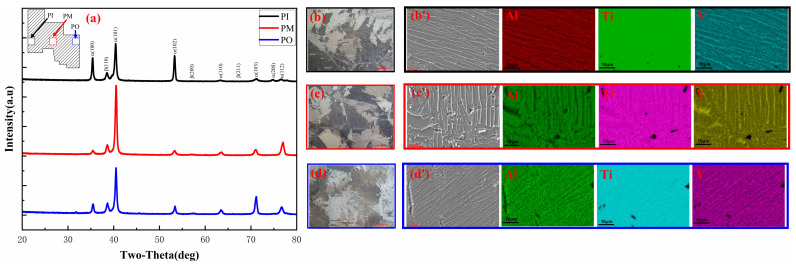
Phase identification and microstructure of the wheel hub prepared according to Level 5: (**a**) XRD patterns; the optical micrographs of (**b**) PI, (**c**) PM, (**d**) PO; SEM images and EDS mapping of (**b′**) PI, (**c′**) PM, (**d′**) PM.

Phase identification and microstructure of the wheel hub prepared according to Level 5 were analyzed in Figure 10. The samples of the wheel hub were divided into three parts: PI (inner), PM (middle), and PO (outside). The wheel hub was mainly composed of *α* and *β* phases (Figure 10a). It was observed that the PM sample had a significantly higher peak intensity compared to the PI and PO samples. The change in peak intensity for the *α* phase could be caused by a difference in the temperature field. The peak in the *β* phase in all three samples did not change significantly. This indicated that the *β* phase was uniformly distributed along the radial direction of the wheel hub [30,31,32].

All three locations of the wheel hub exhibited large *β* grain in Figure 10b–d. During the solidification process, the lath *α* phases precipitated in the *β* grain, as shown in Figure 10b′–d′. It could be observed that the *α* grain orientation of three samples displayed obvious differences. The *α* grain orientation of PI and PO samples were parallel, while the PM sample was different. The lath *α* phase displayed disconnectedness, which would be in favor of improving the mechanical properties of the sample. Moreover, the Ti, Al, and V elements were uniformly distributed on the sample surface for all three samples [33].

In summary, the sample prepared according to Level 5 was composed of *α* and *β* phases and appeared uniform in element distribution. The PM sample exhibited a disconnected lath *α* and uniform *β* phase.

### 3.3. Mechanical Properties

**Figure 11:**
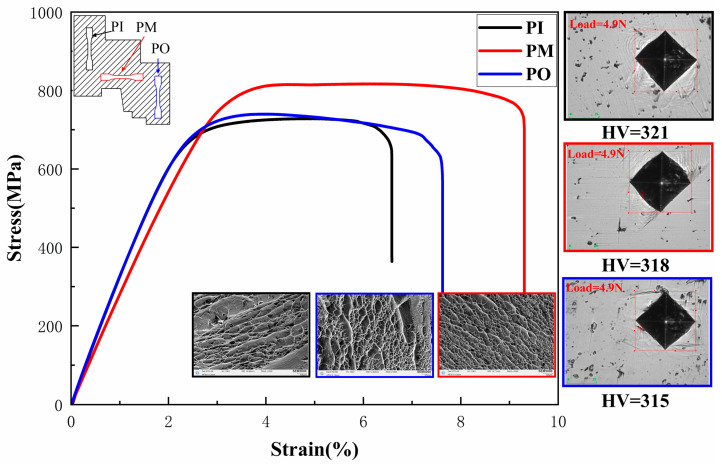
Stress–strain curves, fracture morphology, and hardness of the wheel hub prepared according to Level 5.

The stress–strain curves, fracture morphology, and hardness of the wheel hub, prepared according to Level 5, were analyzed in Figure 11. It could be observed that the tensile strength of the PM sample displayed a maximum value of 820 MPa compared with the PI and PO samples. The reason could be as follows: (1) PI and PO samples were perpendicular to the radial of the wheel hub, while PM samples were parallel to the radial of the wheel hub. (2) The difference in microstructure morphology of the *α* phase. The fracture morphology of the PM sample was dominated by uniformly distributed dimples with similar sizes. These dimples exhibited clear characteristics of ductile fracture. In contrast, the PI and PO samples showed an uneven distribution of dimples and even more tearing edges. Therefore, the tearing edges and nonuniform dimples corresponded to worse mechanical properties. This finding was consistent with the observation that the PI and PO samples had lower mechanical properties compared to the PM sample [34,35].

It was evident that the wheel hub exhibited a relatively uniform distribution of hardness in Figure 11. The hardness values at three different positions were 315, 318, and 321 HV, respectively. Moreover, slip lines close to the hardness indentation appeared, which were induced by compressive indenter (Figure 11). These slip lines were commonly utilized to assess the elastoplastic behavior of materials due to the simplicity of the technique compared with other conventional methods. It could also be observed that wave-shaped slip lines were observed around the edge of indentations for all three samples. It was reported that wave-shaped slip lines appeared around the indentation of the higher hardness samples [36,37]. Thus, the indentations of the three samples corresponded to high hardness.

**Figure 12:**
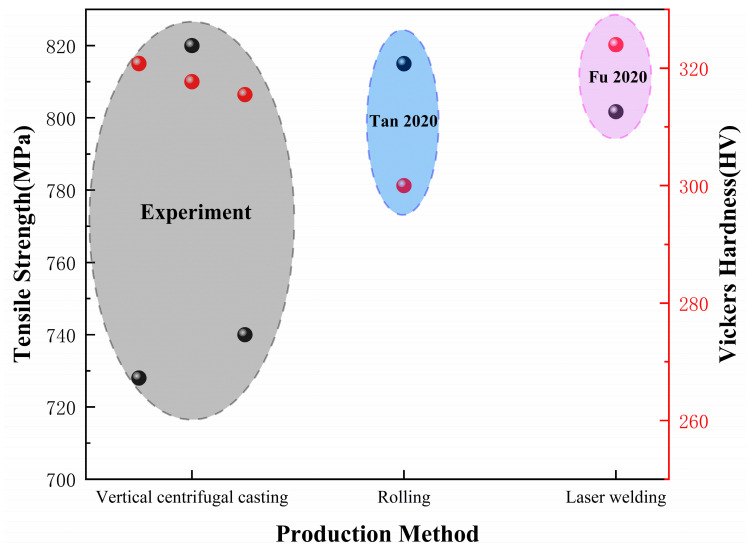
Compared mechanical properties of TC4 wheel hub prepared by vertical centrifugal casting method with that of TC4 alloy prepared by rolling method and laser welding method [38,39].

The mechanical properties of the TC4 alloy prepared by the rolling method [38] and laser welding method [39] were compared with the TC4 wheel hub prepared by the vertical centrifugal casting method in Figure 12. It could be observed that the tensile strength of the vertical centrifugal casting reached 820 MPa, which was higher than that of rolling (815 MPa) and laser welding (800 MPa). Similarly, the Vickers hardness of the TC4 wheel hub (320 HV) was higher than the rolling (300 HV) and close to the laser welding (324 HV) sample. Therefore, the above analysis indicated that the TC4 wheel hub prepared by the vertical centrifugal casting method exhibited excellent mechanical properties.

## 4. Conclusions

The solidification process of the TC4 wheel hub was investigated through ProCAST simulation and an orthogonal experimental design. TC4 wheel hubs were prepared by means of the vertical centrifugal casting method. The following conclusions were drawn:

(1) Nine levels containing three process parameters (rotational speed, pouring temperature, and mold preheating temperature) were designed according to an orthogonal experiment. The rotational speed mainly affected the velocity field, while the pouring temperature and the mold preheating temperature mainly affected the temperature field and solidification time.
(2) According to the orthogonal experiment and extremum difference analysis, the rotational speed (r = 0.07) had the greatest influence on mold defects, followed by the mold preheating temperature (r = 0.06) and finally the pouring temperature (r = 0.03). Therefore, the optimal parameters for the wheel hub were as follows: rotational speed of 225 rpm, mold preheating temperature of 400 °C, and pouring temperature of 1750 °C.
(3) The distribution of defects in the actual wheel hub for Level 5 and Level 6 was consistent with the simulation results, thus validating the effectiveness of the simulation method. The sample prepared according to Level 5 was composed of *α* and *β* phases and had a uniform element distribution. Moreover, the PM sample exhibited disconnected lath *α* and uniformly *β* phases.
(4) Compared with the other samples at different locations, the PM sample revealed a higher tensile strength of 820 MPa and a similar Vickers hardness of 320 HV. Meanwhile, the fracture surfaces of the PM sample were occupied by uniformly distributed dimples. Wave-shaped slip lines at the edge of the indentation corresponded with a high hardness.

However, the grain growth simulation and further analysis of the microstructure of the wheel hub will be executed to further verify the reliability of centrifugal casting in the following research.

## Figures and Tables

**Table 1:** Design of orthogonal experiment.

Level	Rotational Speed (R)/rpm	Mold Preheating Temperature (M)/°C	Pouring Temperature (P)/°C
1	200	400	1730
2	225	450	1730
3	250	500	1730
4	200	500	1750
5	225	400	1750
6	250	450	1750
7	200	450	1770
8	225	500	1770
9	250	400	1770

**Table 2:** Main simulation parameters.

Simulation Parameters (Unit)	Value
The number of 2D elements	33,224
The number of 3D elements	397,642
Interfacial heat transfer coefficient of alloy and outer mold (W·m^−2^·K^−1^)	400
Interfacial heat transfer coefficient of alloy and inner mold (W·m^−2^·K^−1^)	300
Mass flow rate (kg/s)	35
Stop criterion: Final temperature (°C)	400
Maximum fill fraction	100%

**Table 3:** Chemical composition of Ti-6Al-4V (wt.%).

Ti	Al	V	N	O	Fe	H
89.135	6.3	4.25	0.004	0.15	0.16	0.001

**Table 4:** Result of orthogonal experiment.

Level	Rotational Speed (R)/rpm	Mold Preheating Temperature (M)/°C	Pouring Temperature (P)/°C	Score
1	200	400	1730	4.13
2	225	450	1730	4.10
3	250	500	1730	4.17
4	200	500	1750	4.08
5	225	400	1750	4.02
6	250	450	1750	4.20
7	200	450	1770	4.15
8	225	500	1770	4.14
9	250	400	1770	4.11
K1	4.12	4.09	4.13	
K2	4.09	4.15	4.10	
K3	4.16	4.13	4.13	
r	0.07	0.06	0.03	
Order	R > M > P			
Optimization	225	400	1750	

## Data Availability

Data will be made available on request.
